# Microsurgical and Descriptive Three-Dimensional Analysis of the Subarticular Trigone: A Guidemap for Standardizing Lateral Recess Decompression

**DOI:** 10.7759/cureus.62303

**Published:** 2024-06-13

**Authors:** Spyridon Komaitis, Elie Najjar, Mohamed A Hassanin, Daniel D'Aquino, Nasir A Quraishi, Khalid M Salem

**Affiliations:** 1 Spinal Unit, The Centre for Spinal Studies and Surgery, Queens Medical Centre, Nottingham University Hospitals, Nottingham, GBR

**Keywords:** surgical anatomy, anatomy, decompression, subarticular zone, lateral recess

## Abstract

Background

Lateral recess decompression has remained a cornerstone spinal procedure for decades. Despite its popularity, a significant lack of evidence in the literature exists concerning microsurgical anatomy and pertinent surgical landmarks, resulting in non-standardized nomenclature, descriptions, and surgical approaches.

Objective

This study provides an in-depth microsurgical and descriptive analysis of the subarticular trigone (SAT), serving as an anatomical guide and a tool to foster consistency in nomenclature and standardization of surgical approaches.

Methods

We analyzed 35 high-resolution lumbar spine CT scans, employing three-dimensional (3D) processing techniques. The SAT is introduced to delineate the bony prominence enveloping the superiomedial quadrant of the pedicle. The SAT encompasses two zones: (1) a superior zone above the superior pedicular line, corresponding to the medial part of the body of the ascending facet (AF), and (2) an inferior zone between the superior and middle pedicular lines, corresponding to the root of the AF and the medial pars/superior lamina. The superior subarticular point (SSP) and medial subarticular point (MESP) serve as key reference landmarks. The SAT forms the roof of the lateral recess and the region requiring resection during decompression of the traversing root in this anatomical corridor. Various measurements, including SSP and MESP to lateral pars, tip of the facet and spino-laminar junction distance, mean width of the sublaminar ridge (SLR), and percentage of the facet that requires resection for adequate SAT decompression, were carried out.

Results

The mean distance of the SSP to the lateral pars ranges from 7 to 9.2 mm, to the tip of the descending from 9.3 to 10.1 mm, and to the spino-laminar junction from 6.7 to 8.1 mm. The MESP is located at a mean distance of 5.4-6.9 mm from the medial pedicular line. The mean width of the SLR varies from 18.6 to 29.4 mm. Finally, the percentage of total facet width that needs to be removed to adequately decompress the SAT extends from 32% at L4 to 36% at L1.

Conclusions

This study presents comprehensive insights into the surgical, descriptive, and correlative anatomy of the lateral recess, emphasizing the SAT. The extrapolated data offer a framework for achieving uniformity in surgical planning and advocate for standardized nomenclature.

## Introduction

The earliest documented instances of lumbar stenosis are traceable to the seminal works of Sachs and Fraenkel during the waning years of the 20th century [1]. Williams and Yglesias stood among the pioneering figures in the delineation of the so-called facet syndrome, as elucidated in their 1933 publication [2]. In subsequent decades, various authors underscored the clinical significance of degeneration and hypertrophy of the superior articular process, emphasizing its role as a structural instigator of nerve root compression.

Within this contextual framework, Epstein et al. presented a case series, scrutinizing patients experiencing sciatica attributable to nerve root compression within the anatomical region identified as the lateral recess or subarticular gutter [3]. Since then, an expanding body of literature has probed diverse surgical modalities employed for the decompression of this intricate region, with minimally invasive and endoscopic techniques emerging as noteworthy contributors in recent years [4-9].

According to conventional characterization, the lateral recess denotes an anatomical passageway circumscribed by the pedicle laterally, the superior articular process and ligamentum flavum posteriorly, and the posterior wall of the vertebral body and disc anteriorly [10,11]. Despite lateral recess decompression being one of the most frequently employed spinal procedures in the realm of degenerative diseases, a lack of evidence in the literature pertaining to the microsurgical, topographical, and correlative anatomy of the lateral recess exists. This results in a non-standardized surgical approach to the region, particularly for inexperienced surgeons, characterized by imprecision, a lack of uniformity, and a deficit in repeatability.

Against this backdrop, the present study endeavors to furnish a comprehensive descriptive anatomical analysis of the lateral recess, employing standardized nomenclature. Furthermore, it seeks to delineate a microsurgical roadmap of key landmark structures necessitating identification in a stepwise manner during approaches to this area, thereby ensuring an efficacious decompression. Comprehension of the microsurgical anatomy and intricate anatomical interrelations among the aforementioned structures holds the potential to contribute to a standardized surgical technique, mitigate the risk of under- or over-decompression, and concurrently assure repeatability for the surgeon.

## Materials and methods

This study enlisted 35 high-resolution CT scans, comprising 18 male and 17 female subjects aged between 25 and 40 years. These individuals were devoid of significant degenerative changes, trauma, or other pathologies in the lumbar spine and were scanned as part of other screening protocols. As an additional precautionary measure to safeguard patient confidentiality, all Digital Imaging and Communications in Medicine (DICOM) files underwent anonymization utilizing DICOM Cleaner (PixelMed, Nordrhein-Westfalen, Germany) software before undergoing processing by the authors. This approach ensured the protection of personal data and the preservation of anonymity for all subjects involved.

The DICOM files were subsequently subjected to processing through three-dimensional (3D) processing and segmentation software (3D Slicer version 4.8.1, Texas, USA). The ensuing 3D models were generated for each scan, and meticulous processing enabled visualization from diverse angles and perspectives, thereby facilitating a comprehensive inspection of the designated areas of interest. The investigative focus included various measurements conducted on a decimal millimeter scale, concentrating on the correlative anatomy of the anatomical structures delineating the lateral recess. Three authors (SK, EN, and MH) proceeded with various measurements and the mean value of three measurements in cases of small interobserver variability. These measurements mainly investigated the distance of the superior subarticular point (SSP) and medial subarticular point (MESP) from various key landmarks, including the lateral pars, the tip of the descending facet (DF) (DF is recruited throughout this manuscript to describe the inferior articular process), the spino-laminar junction, and facet total width. Simulations of bony decompression on the 3D model were executed, extending to the predetermined landmarks, followed by measurement of the width of the facet removed and comparison to the total facet width to calculate the percentage of facet removal required for adequate decompression. Various illustrations of the relevant anatomy are offered, with special emphasis on the anatomy of the subarticular trigone (SAT).

## Results

**Figure 1:**
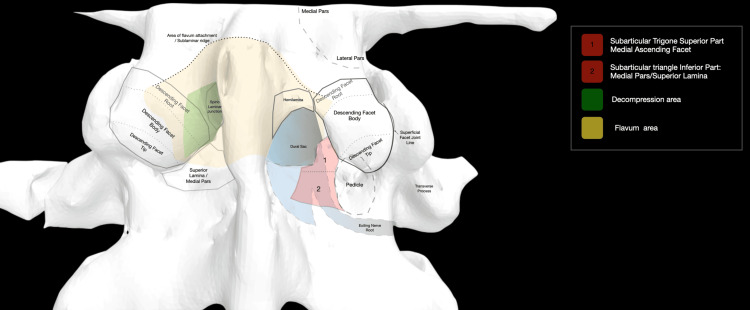
Superficial anatomy and deep anatomy are superimposed. On the right side, a medial facetectomy and laminotomy are simulated, exposing the SAT and its two zones SAT: subarticular trigone Image Credit: Spyridon Komaitis

**Figure 2:**
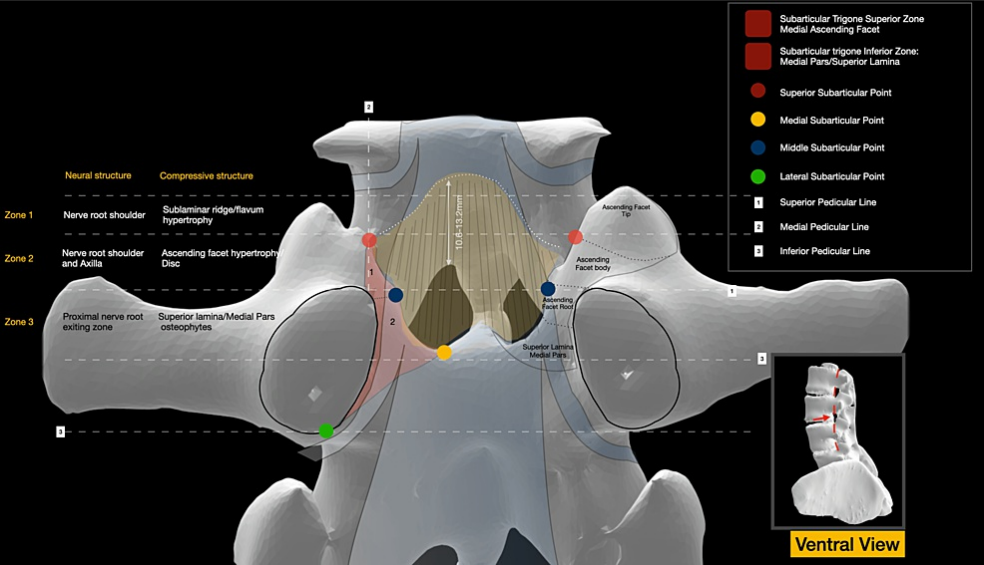
Ventral view of a CT 3D reconstruction of the roof of the spinal canal. The anatomy of the SAT as well as the various anatomical landmarks, including the SSP, MESP, and LSP, are illustrated. The zones of the lateral recess with related compressive structures are also presented CT: computed tomography, 3D: three-dimensional, SAT: subarticular trigone, SSP: superior subarticular point, MESP: medial subarticular point, LSP: lateral subarticular point Image Credit: Spyridon Komaitis

The SAT is identified as the triangular osseous prominence situated in a deep anatomical relation to the body and tip of the inferior articular process (Figures 1-2).

**Figure 3:**
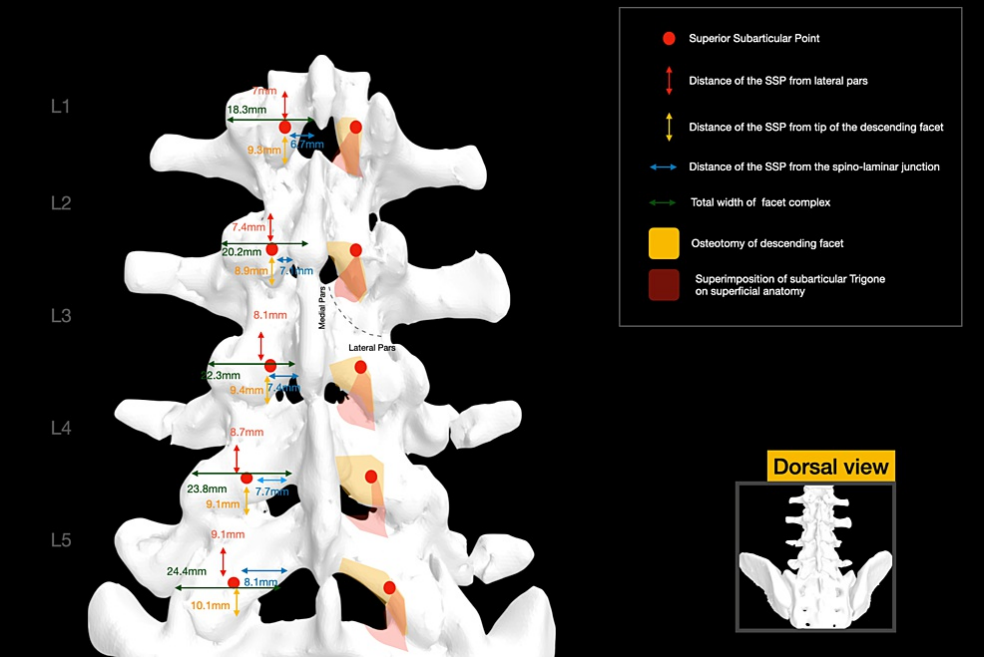
Superimposition of the anatomy of the SAT on the superficial level. Mean values for various measurements are illustrated for each level SAT: subarticular trigone Image credit: Spyridon Komaitis

**Figure 4:**
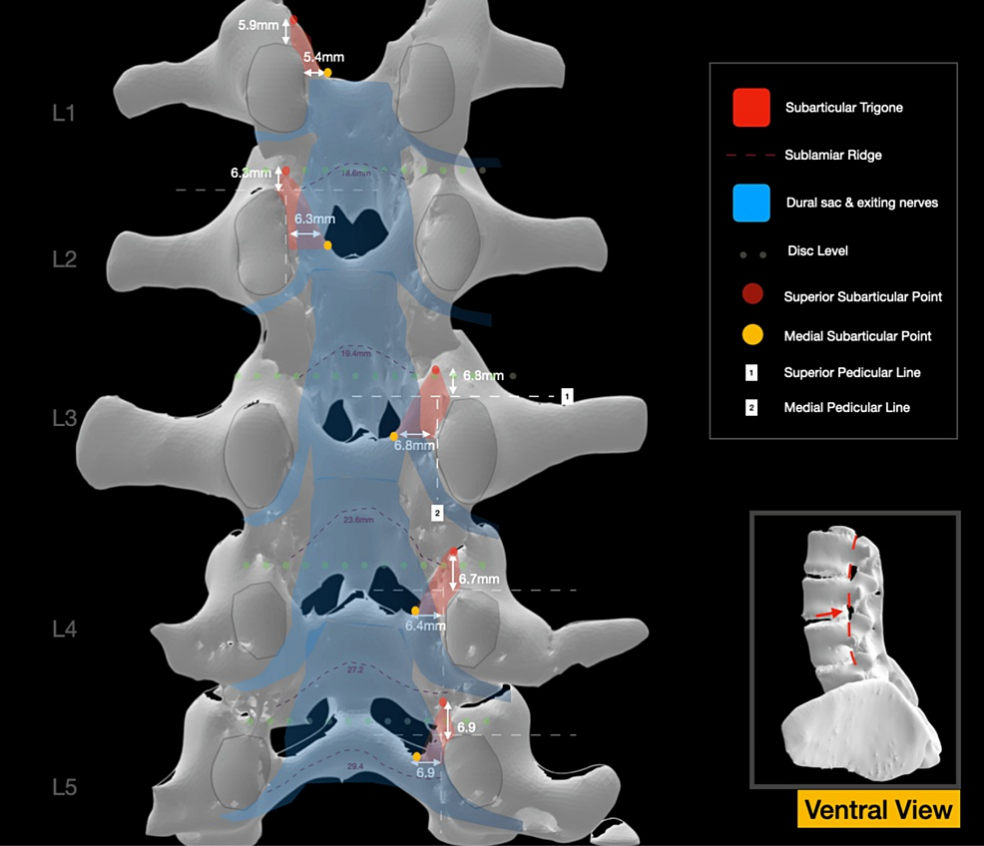
Ventral view of the roof of the spinal canal. The anatomy of the SLR is illustrated. The mean values of the distances of the SSP to SPL and the MESP to MPL are presented for each level. The relevant position of the thecal sac and nerve roots is also illustrated SSP: superior subarticular point, SPL: superior pedicular line, SLR: sublaminar ridge, MPL: medial pedicular line, MESP: medial subarticular point Image Credit: Spyridon Komaitis

Encompassing the superomedial and inferomedial quadrants of the pedicle, the SAT is defined by three key anatomical points: the SSP designates the intersection of the medial pedicular line (MPL), a vertical line tangential to the medial facet wall, with the superior articular process (described as ascending facet (AF) throughout this manuscript; Figure 2). The SSP exhibits a mean distance ranging from 5.9 to 6.9 mm from the superior pedicular line (SPL), contingent upon the vertebral level. Its mean vertical distance from the lateral pars ranges between 7 and 9.2 mm, from the tip of the DF between 9.3 and 10.1 mm, and from the spino-laminar junction between 6.7 and 8.1 mm, respectively (Figures 3-4).

**Figure 5:**
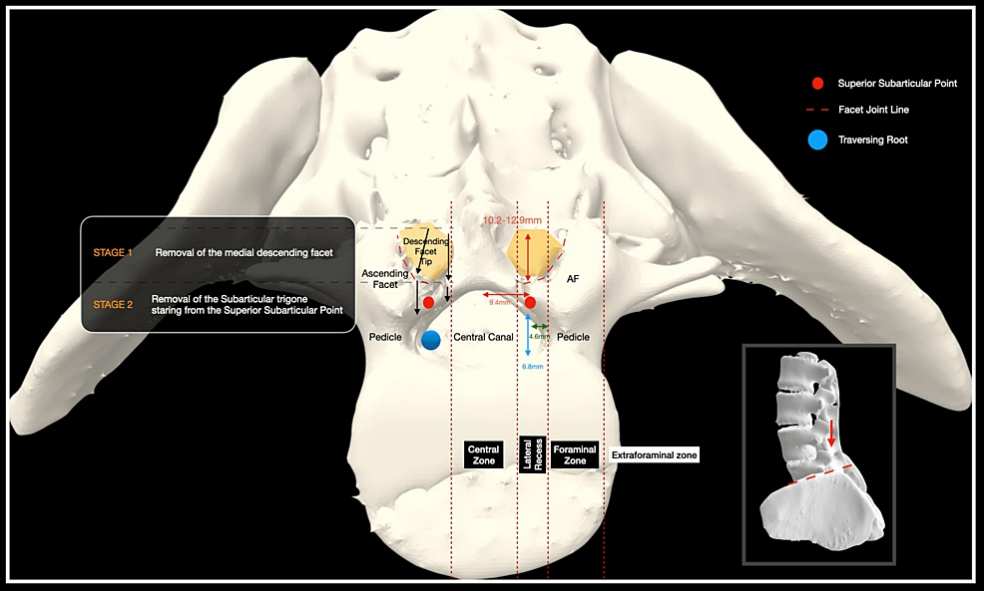
Axial cross-section of the lateral recess and related structures at the level of L5 AF: ascending facet Image Credit: Spyridon Komaitis

Typically, the SSP is situated at a depth of 10.8 to 12.3 mm from the surface of the DF (Figure 5).

The MESP corresponds to the intersection of the MPL, a horizontal line intersecting the ninth and third hours of the pedicle, with the superior lamina (Figure 2). The MESP, integral to the delineation of the SAT, is positioned at a mean distance of 5.4 to 6.9 mm from the MPL (Figure 4).

**Figure 6:**
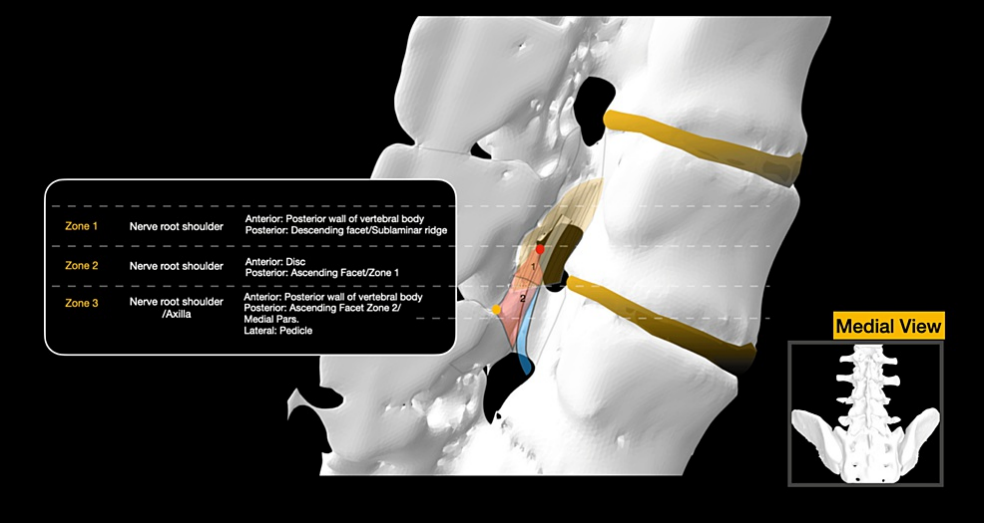
Midsagittal cross-section of the central canal, allowing visualization of the SAT. The anatomy of the traversing nerve root is superimposed. The flavum attachment area is also highlighted in yellow SAT: subarticular trigone Image Credit: Spyridon Komaitis

The lateral subarticular point (LSP) designates the sixth hour of the pedicle, while the MESP is positioned at the intersection of the SPL with the root of the AF (Figure 2). The SPL divides the SAT into two discernible zones: Zone 1 (Z1), located above the SPL, encompasses the medial portion of the AF body, while Zone 2 (Z2), situated below the SPL, includes the root of the AF as well as the medial pars/superior lamina (Figures 1-2, Figure 6).

Imaginary horizontal lines traversing the superior articular point (SAP) and MESP delineate the AF into three zones: the root (below the MESP level), the body (between the MESP and SAP), and the tip (above the SAP) (Figure 2).

The sublaminar ridge (SLR), corresponding to the bony impression formed by the attachment of the ligamentum flavum, exhibits a mean width ranging from 18.6 mm at the level of L1 to 29.4 mm at the level of L5. Extending laterally to the body of the DF, the SLR typically terminates within 2-3 mm cranially or caudally to the SAP and is located 10.6-13.2 mm above the inferior border of the lamina (Figure 2). The total width of the facet complex ranges from a mean of 18.3 mm at the level of L1 to a mean of 24.4 mm at the level of L5 (Figure 3).

The traversing nerve root navigates three potential compression zones before exiting below the pedicle. A superior zone, deep in the SLR, is susceptible to flaval hypertrophy-induced compression of the nerve root shoulder. A middle area, located deep in Zone 1 of the SAT, may experience compression of the nerve root shoulder due to hypertrophy of the AF or disc herniations. An inferior zone, corresponding to Zone 2 of the SAT, where osteophytes of the medial pars/lamina or caudally migrated discs, may impact the axilla before the root enters the foraminal area (Figure 2). A summary of the various measurements is presented in Table 1 and also illustrated in Figures 3-4.

**Table 1:** Various measurements focusing on the anatomy of the SAT FW: facet width, LP: lateral pars, MPL: medial pedicular line, MESP: medial subarticular point, SAT: subarticular trigone, SLJ: spino-laminar junction, SLR: sublaminar ridge, SPL: superior pedicular line, SSP: superior subarticular point, TDF: tip of descending facet, DF: descending facet

	SSP to LP distance (mm) SSP/LP mean (range)	SSP to TDF distance (mm) SSP/TDF mean (range)	SSP to SLJ distance (mm) SSP/SLJ mean (range)	SSP to SPL distance (mm) SSP/SPL mean (range)	MESP to MPL distance (mm) MESP/MPL mean (range)	Total width of SLR (mm) SLR mean (range)	Total FW (mm) FW mean (range)	Depth of DF to SSP (mm) mean (range)	Percentage of total FW removed to decompress SAT mean (range)
L1	7 (6.8-7.2)	9.3 (8.9-9.7)	6.7 (6.0-6.9)	5.9 (5.6-6.1)	5.4 (5.1-5.5.9)	18.6 (17.5-19.2)	18.3 (17.6-19.9)	12.3 (10.9-12.9)	36%
L2	7.4 (7.2-7.6)	8.9 (8.4-9.3)	7.1 (6.2-7.3)	6.3 (5.6-6.1)	6.3 (5.7-6.9)	19.4 (18.5-20.4)	20.2 (19-22.5)	11.9 (11.2- 12.7)	35%
L3	8.7 (7.3-8.9)	9.4 (8.8-9.9)	7.4 (6.5-7.6)	6.8 (6.0-7.3)	6.8 (6.2-7.4)	23.6 (22.2-26.0)	22.3 (20.5-25.3)	11.7 (10.7-12.8)	33%
L4	9.1 (8.9-9.3)	9.1 (8.6-9.4)	7.7 (6.9-8.2)	6.7 (6.1-7.5)	6.4 (5.8-6.7)	27.2 (24.8-29.7)	23.8 (22-26.34)	10.8 (10.1-11.9)	32%
L5	9.2 (8.9-9.4)	10.1 (9.2-10.6)	8.1 (7.7-8.6)	6.9 (6.3-7.6)	6.9 (6.5-7.4)	29.4 (27.8-31.9)	24.4 (22.5-26.8)	11.4 (10.2-12)	33%

## Discussion

The earliest instances of root compression resulting from disc herniation can be traced back to the seminal works of Mixter and Barr [12]. Subsequent years saw various authors recognize the potential for nerve root compression even in the absence of disc material within the canal, leading to the gradual development of the concept of lateral recess compression. The advent of imaging revolution modalities, such as radiculography and later, computerized tomography or myelography, provided innovative insights [13].

Studies conducted in the 1980s and early 1990s by researchers like Ciric et al., Matozzi et al., and Wilmink played a crucial role in delineating the lateral recess as a distinct anatomical locus [11,14,15]. They identified different patterns of compression and furnished radiological measurements that contribute to defining the presence of compression in this region. The introduction of MRI further enhanced our diagnostic capabilities and sensitivity in identifying pathologies associated with the lateral recess [16-18].

Pure anatomical descriptions of the lateral recess area remain limited, with the notable exception of a landmark study by Lee et al. [10]. This study identified and described three zones within the lateral lumbar region, namely the entry zone, the mid-zone, and the exiting zone, standing out as noteworthy contributions to our understanding of this anatomical region.

In recent decades, compression of the traversing nerve root within the subarticular gutter has emerged as one of the most prevalent degenerative pathologies treated by spinal surgeons. Addressing such compressions involves a range of treatment modalities, spanning from nerve root injections and open lateral recess decompressions through unilateral selective approaches to minimally invasive techniques and more recently endoscopic approaches [4,5,7,8,19,20]. Alongside a gross anatomical delineation of the lateral recess, a microsurgical comprehension of the involved region is imperative to ensure thorough decompression without extensive facet/pars damage that would lead to instability, as well as procedural reproducibility, thereby mitigating the learning curve associated with these techniques.

In this present study, we introduce, for the first time in the literature, a microsurgical perspective on the subarticular zone, revisiting its anatomy and nomenclature, and establishing specific landmarks to guide surgical decompression, including the SSP and the MESP. The SAT stands prominent in the understanding of this area and represents the bony roof that needs to be decompressed to effectively relieve the traversing nerve root, irrespective of the presence or absence of disc pathology. This medial facetectomy procedure is well known empirically, but no specific micro-surgical anatomical key points are available in the literature to guide a stepwise decompression. In this context, the current study serves by offering a well-standardized nomenclature and a step-wise guide to guarantee repeatability and adequate, microsurgical-anatomy-guided decompression of the subarticular zone.

Surgical consideration and significance of anatomical landmarks: a stepwise surgical anatomy-based approach

The initiation of lateral recess decompression involves a precise 2-3 mm drilling of the medial root, body, and tip of the DF, extending medially to the spino-laminar junction. The cephalad extension of the drilling is determined by identifying the tip of the DF and subsequently guiding the decompression 8.9 to 10.1 mm cranially. In this very region of the DF body, meticulous drilling exposes the joint surface, the medial silhouette of the AF, and the SSP. At this juncture, a curette can be employed to detach the flavum attachment from the SSP undersurface of the AF. Subsequently, a 2 mm Kerrison rongeur can be utilized to commence the resection of Z1 of the SAT, starting from the SAP and progressing caudally. This maneuver reveals and releases the shoulder of the nerve root.

Continuing the decompression of Z1, the Kerrison is advanced caudally until it contacts the medial wall of the facet. A curette is then employed to detach the inferior flaval attachment from the superior lamina/medial pars area. Subsequently, a 2 mm Kerrison is reintroduced to continue the decompression below the SPL. Medially, decompression should extend as far as the MESP to widely expose the axilla of the nerve root. Caudally, the medial border of the pedicle is carefully followed and palpated using a blunt hookup to the LSP. Reaching the LSP is signified by the hook's entrance into the foraminal area.

Following the completion of these steps, the cranial decompression area should always be revisited, and a meticulous undercutting of the SLR, 2-3 mm cranial to the SAP, should always be pursued. Early identification of the SSP serves to prevent cranial or lateral overextension of the decompression, mitigating the risk of intra- or postoperative pars fracture and subsequent iatrogenic instability. Conversely, this proactive identification ensures thorough decompression of the nerve root's shoulder. The extension of decompression medially, reaching as far as the MESP, accomplishes the exposure of the axilla of the nerve root, a region prone to compression, particularly in the presence of sequestrated disc segments or laminar osteophytes.

Caudal extension, extending as far as the LSP, guarantees the comprehensive decompression of the root proximal to its exiting zone, specifically in cases of SLR enthesopathy or flaval hypertrophy. Previous studies advocate that achieving lateral recess decompression requires a facetectomy ranging from one-third to half of the facet joint [6,21,22]. As elucidated in Table 1, confining the decompression within the boundaries of the SAT as presented in the current study ensures maintaining facet resection to around one-third of its total width and therefore minimizes the risk of requiring future fusion due to iatrogenic facet failure and instability.

In the presence of disc pathology, after completing the bony decompression of the SAT, a blunt hook combined with bipolar coagulation of epidural veins can be utilized to untether the epidural space in the area of the SSP; in the same area, a nerve root retractor can be inserted to initiate retraction of the nerve root. Starting the nerve root retraction cranially, in the area of the SSP, minimizes initial traction while the nerve root is still tethered to the epidural space and reduces the risk of nerve root injury.

Limitations of the study

The present study employs 3D processing of CT. While the spatial resolution attained is notably high, it is pertinent to acknowledge that its precision may, at times, be comparatively suboptimal when contrasted with cadaveric studies. Furthermore, certain measurements, particularly those surrounding the mobile facet joint, may be susceptible to a modest margin of error. This potential discrepancy arises from the supine positioning of the body during image acquisition, in contrast to the prone surgical positioning that is typically assumed. Finally, all the investigated 3D models recruited in this study were devoid of degenerative changes. Anatomy of the degenerative spine can significantly vary; however, even in the case of facet arthropathy or in the presence of osteophytes, the main anatomical landmarks can still be recognized and therefore can guide decompression.

## Conclusions

The lateral recess is a commonly implicated site for nerve root compression, demarcated by distinct anatomical structures, namely the superior articular process, intervertebral disc, posterior vertebral wall, and medial pars. The present investigation contributes a meticulous surgical anatomy analysis of this region, introducing critical landmarks such as the SAT, SSP, MESP, and LSP. These landmarks serve as guideposts for surgical procedures, fostering a standardized approach to surgical interventions. Additionally, the study presents various anatomical measurements designed to facilitate precise surgical decompression. This stands as one of the limited studies in the relevant literature focused on the microsurgical anatomy of the lateral recess. Its emphasis on delineating key anatomical features and providing practical metrics for surgical interventions contributes to a deeper understanding of the intricacies within this anatomical domain.
